# Stress increases periodontal inflammation

**DOI:** 10.3892/etm.2012.675

**Published:** 2012-08-20

**Authors:** CÉSAR RIVERA, FRANCISCO MONSALVE, IVÁN SUAZO, JAVIERA BECERRA

**Affiliations:** 1 Unit of Histology and Embryology, Department of Basic Biomedical Sciences, Faculty of Health Sciences;; 2 Master Program in Biomedical Sciences, Oral Pathology mention, University of Talca, Talca;; 3 Faculty of Medicine, Diego Portales University, Santiago;; 4 School of Dentistry, Faculty of Health Sciences, University of Talca, Talca, Chile

**Keywords:** stress, gingiva, inflammation, periodontal disease, periodontal tissues, periodontium, stress analysis

## Abstract

This study aimed to examine the effect of chronic restraint stress (RS) on the severity of experimental periodontal disease in rats. A total of 32 male Sprague Dawley (SD) rats were divided into four groups: i) Rats receiving two treatment regimens, chronic stress induced by movement restriction in acrylic cylinders for 1–1.5 h daily and induction of experimental periodontal disease, using a nylon ligature which was placed around the first left mandibular molars (n=8); ii) induction of periodontal disease, without RS (n=8); iii) RS (n=8) and iv) control (n=8). After 15 days, blood samples were obtained, and blood glucose levels and the corticosterone concentration were measured as stress markers. The severity of periodontal disease was analyzed according to the level of gingival and bone inflammation, leading to compromise of the teeth involved. Chronic stress was induced with movement restriction (P≤0.05, Mann-Whitney U-test) and increased the severity (P≤0.05, Mann-Whitney U-test) of experimental perio dontal disease in rats, according to the level of gingival and bone inflammation around the first left mandibular molars. The results of the present study showed that RS modulates periodontal inflammation and that the rat model described herein is suitable for investigating the association between stress and periodontal disease.

## Introduction

The development of periodontal inflammation is a complex process, therefore animal models have been developed to assist in its understanding. Experimental periodontitis induced by the placement of nylon or cotton ligatures around molars (allowing the retention of plaque), is one of the most widely used models (1–4).

The main etiological factor of periodontal disease is bacterial plaque, but the pathogenesis of the disease is affected by environmental factors that modify or induce systemic progression, such as stress (3,5,6).

Human studies suggest that negative life events and psychological factors may contribute to an increased susceptibility for periodontal disease (5,7–10).

It has been reported that stress produces neuroendocrine changes and certain adverse effects on the immune system, which affect the inflammatory response on periodontal tissues (11,12).

The restriction movement technique has become a standard procedure to study stress effects, particularly when using rodents as study subjects (13,14). This model has been previously used to associate chronic exposure to stress and periodontal destruction (1–3,15,16).

Studies on animals indicated that chronic stress may modulate pathophysiological states of inflammation, causing an accelerated degradation of periodontal tissues (1).

Despite the accumulated evidence, a direct association between periodontal disease and stress is not entirely clear (17). In addition, there are no previous studies or investigations that consider the effect of habituation in study animals subjected to chronic stress. This factor should be considered in the design and research methodology, as the physiological response obtained may not represent the reality (18,19).

Therefore, the purpose of this study was to clarify the role of chronic stress on the severity of experimental periodontitis in rats, taking into account previously unconsidered issues.

## Materials and methods

### Experimental design

An experimental design of randomized blocks was used. The independent variable was stress exposition and the dependent variable was periodontal disease severity. All procedures followed the guidelines of the Guide for the Care and Use of Laboratory Animals, National Research Council (20) and were approved by the Bioethics Committee of the University of Talca (Talca, Chile).

### Animals

A total of 32 male Sprague Dawley (SD) rats of 12 weeks of age (330–430 g) were used. No blood relatives, with appropriate health certificates, were obtained from the Institute of Biomedical Sciences (ICBM, University of Chile). The rats were kept under controlled temperatures (22±1°C), under 12-h light/dark (the light was turned on at 08:00 am) conditions, with freely available food and water, in groups of four rats in polycarbonate enclosures enriched with tissue paper and cardboard rolls (21), at the Animal Facility of the University of Talca.

**Figure 1:**
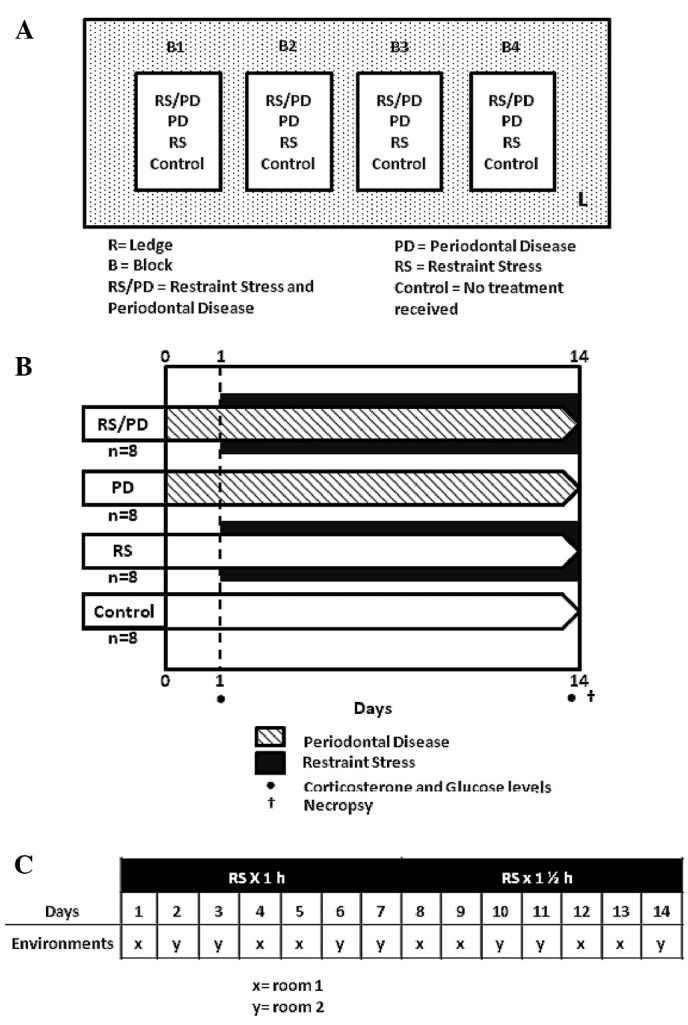
(A) Distribution of the experimental units. Each cage represented a block. (B) Relevant aspects of the pilot phase; distribution of groups according to treatment received is shown. (C) Movement restriction protocol is shown. Exposure to restriction of movement begins at day 1 (RS), 1 h/day the first week; then, from day 8, RS is performed for 1.5 h, in order to complete the second week, in different environments, represented by x and y.

Rats were divided into four groups (Fig. 1A): the RS/PD group (n=8) received stress by movement restriction and periodontitis; PD group (n=8) received stress-induced periodontitis; RS group (n=8) received only stress and the control group (n=8) received no treatment at all.

### Induction of experimental periodontal disease

**Figure 2:**
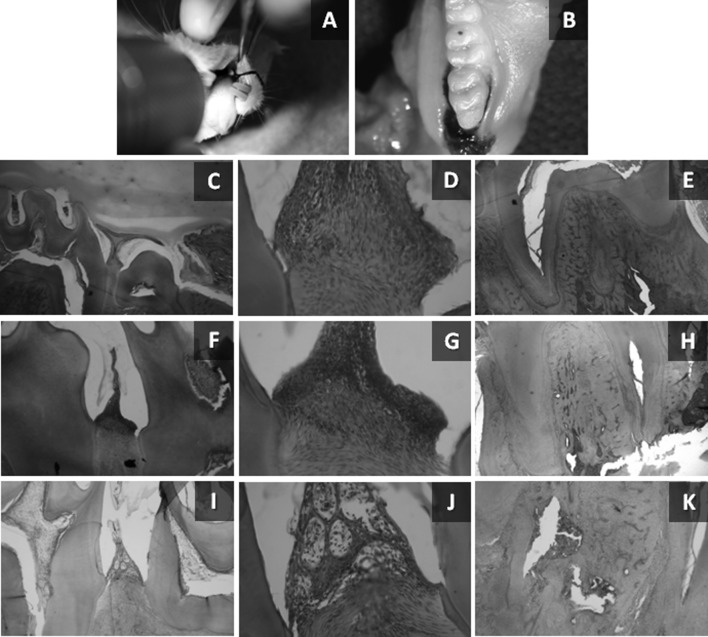
(A) Periodontal disease (PD) induction by molar ligature. (B) Intraoral appearance at the end of the experimental phase. (C) Control group gingival papilla remains intact. (D) No bone resorption and (E) absence of gingival inflammatory infiltrate. (F) The PD group presented integrity loss of the gingival papilla, (G) inflammatory infiltrate in gingival epithelium and connective tissue. (H) Areas of interradicular bone resorption are shown. (I) The RS/PD group shows loss of integrity of the gingival papilla. (J) Large inflammatory infiltrate in gingival epithelium and connective tissue, there is also a loss in the continuity of the collagen fibers and (K) large irregular areas of alveolar bone loss, sometimes replaced by hematopoietic and hyperplastic bone marrow.

Significant events of the pilot phase are shown in Fig. 1B. Prior to the intraoral procedures, the rats were anesthetized with 10% ketamine/2% xylazine/1% acepromazine (Drag Pharma Chile Invetec S.A., Santiago, Chile) at a ratio of 50/5/1 mg/kg intramuscularly (IM). Having established anesthesia, we applied a ligature (4-0, Ethicon, Johnson & Johnson Company) around the neck of the left mandibular first molars (M1s) from each rat (Fig. 2A and B). The rats remained with ligatures throughout the experimental period of 15 days (to allow retention of plaque). Each day the correct position of the bands was confirmed.

### Restraint stress (RS) model

From day 1 of the pilot phase, animals were stressed 1 h/day for the first 7 days, and then 1.5 h in the following 7 days, in two different environments. The animals were alternated to avoid habituation to the stressor stimulus and environment (Fig. 1C) in properly ventilated acrylic cylinders (60 mm in diameter). Each session was undertaken for 1–1.5 h between 08:00 and 12:00 am, during which rats were fasted from food or water.

### Laboratory assays

After the first and last cycle of restriction of movement, plasma samples were obtained from the leg vein of each subject and stored at −20°C, until determination of plasma levels of corticosterone and glucose in the Laboratory of Animal Physiology and Endocrinology (University of Concepción, Chillán, Chile). Corticosterone was quantified by means of a commercial ELISA kit (DRG International, Austin, TX, USA), validated for rat corticosterone, with an intrassay coefficient of 3%. Glucose was determined with a kit (Roche, Mannheim, Germany) based on the GOD-POD method (glucose oxidase and peroxidase) and was measured at 505 nm using a spectrophotometer (Thermo Electron Co., Vantaa, Finland).

### Tissue preparation

After the experimental phase, the animals were sacrificed with an overdose of anesthetic. Immediately thereafter, the mandible was hemisected (two halves by a cut between the lower incisors) and fixed in 10% neutral-buffered formalin. The decalcified tissue blocks were maintained in 5% nitric acid for 7 days after conventional histopathology (H&E staining).

### Incidence

Determination of periodontal disease by histopathological examination was established with a conventional technique of 112 plates of H&E-stained tissues, which were obtained from the mandible processing preparations. The plates were analyzed by an academic (CR) from the University of Talca. The observer was unaware of the group to which the study samples belonged (single-blind model). The incidence of periodontal disease was established by the presence of inflammation or destruction of periodontal tissues.

### Severity

**Figure 3:**
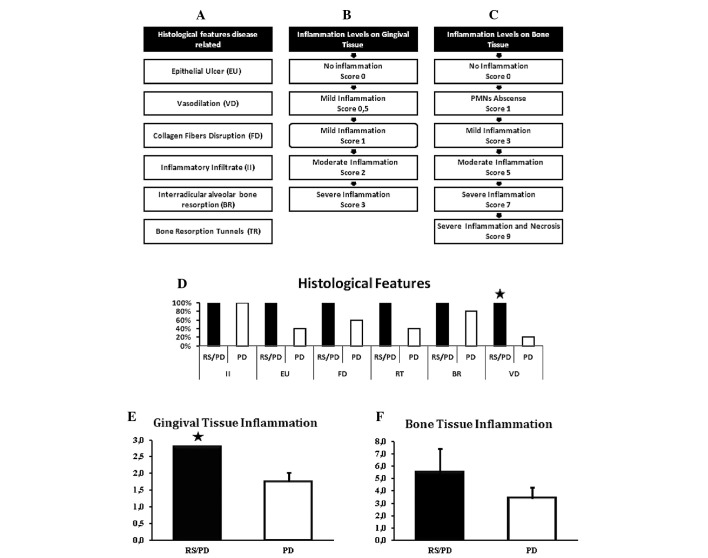
(A) The severity of experimental periodontal disease (PD) was determined. Associated histological features with periodontal disease [modified from Liu *et al* (23) and Garcia (22)] are shown: epithelial ulceration, lack of continuity of the gingival epithelium, vasodilation, increased luminal diameter blood vessels, disrupted collagen fibers, continuity of loss of collagen fibers in connective tissue, presence of inflammatory infiltrate, groups of lymphocytes observed in field; interradicular alveolar bone resorption, continuity loss of interradicular bone, replaced by connective tissue or other; tunnels in bone resorption, osteoclastic resorption in depth areas. (B) Degree of inflammation in the gingival tissues [modified from Liu *et al* (24) and Luan *et al* (25)] is shown: no inflammation, no presence of inflammatory cells (score 0), mild inflammation (score 0.5), limited inflammation of the epithelium, mild inflammation (score 1), inflammation of the connective tissue near the epithelium, with 2–4 inflammatory cells/field; moderate inflammation (score 2), inflammation of the tissue with 5–10 inflammatory cells/field; severe inflammation (score 3), inflammation in the connective tissue consistent with an abscess. (C) Degree of inflammation in bone tissue [taken from Graves *et al* (26)] is shown. The scale was used according to the number of PMNs at the center of the inflammatory infiltrate (1, no PMNs; 3, slight infiltrate; 5, moderate infiltrate; 7, severe infiltrate; and 9, severe infiltrate with cell necrosis). (D) Presence of histological features associated with periodontal disease (percentage) are shown. It is noted that all features are present in 100% of animals in the RS/PD group, which is significantly different to PD in vasodilation (significant difference for Chi-square test, P≤0.05). (E) Restriction of movement increases inflammation of gingival tissue in rats with periodontal disease. Rats in the RS/PD group have an average value of 2.8 (moderate to severe inflammation). Rats in the PD group presented a mean value of 1.8 (mild to moderate inflammation). The difference among the groups was statistically significant (P=0.001 for Student’s t-test). (F) Inflammation of bone tissue. Rats in RS/PD group had an average value of 5.5 (moderate to severe inflammation). The rats in the PD group presented a mean value of 3.4 (inflammation of mild to moderate type). The difference among the groups was not statistically significant (P= 0.064 for Student’s t-test). PMNs, polymorphonuclear leukocytes.

To establish the severity of the disease in the periodontal tissues of the M1s, we examined the presence or absence of pathological histology, according to the parameters of Garcia (22) and Liu *et al* (23) (Fig. 3A). The degree of inflammation in the gingival tissue (24,25) (Fig. 3B) and bone (26) (Fig. 3C) was also determined.

### Statistical analysis

Qualitative data were analyzed by the Chi-square test with Pearson’s correlation. Quantitative data were assessed using the Mann-Whitney U test and Student’s t-test. P≤0.05 was considered to indicate a statistically significant result.

## Results

### SD rats treated with RS had higher levels of plasma corticosterone

Table I shows plasma corticosterone (ng/ml) and glucose (mmol/l) levels. Results of the Mann-Whitney U test established significant differences between the groups treated with RS (RS/PD and RS), compared with final measurements of corticosterone in the groups not treated with RS (PD and control; P≤0,05). There were no statistical differences in the measured glucose levels.

### Histopathological findings

Fig. 2C–K shows the main histological aspects observed in this investigation.

### Incidence of experimental periodontal disease

All the rats treated with molar ligation (RS/PD and PD groups) had inflammation in periodontal tissues (gingival or bone). There was no periodontal inflammation in the untreated animals.

### Severity according to the presence of histological features associated with periodontal disease

RS increased the presence of features associated with periodontal disease (Fig. 3D). Rats in the RS/PD group tended to exhibit greater disruption of collagen fibers of connective tissue, epithelial ulcers, resorption tunnels, interradicular alveolar bone resorption and vasodilation, compared with the PD group, with a statistically significant difference only for vasodilation (P<0.05 for the Chi-square test).

### Severity of periodontal disease according to the degree of gingival inflammation

RS increased the severity of gingival inflammation (Fig. 3E). Rats in the RS/PD group showed an average value of 2.8 inflammatory infiltrate, corresponding to moderate to severe inflammatory process. The PD group demonstrated a value of 1.8, indicating mild to moderate inflammation. To evaluate the difference we used the Student’s t-test, which found a statistically significant difference (P=0.001).

### Severity of periodontal disease according to the degree of bone inflammation

RS did not increase the severity of inflammation in bone marrow (Fig. 3F). Rats in the RS/PD group tended to have a score value of 5–7 points (average 5.5), corresponding to moderate to severe inflammatory process. PD group rats presented with score values of 3–5 points (mean 3.4), indicating a mild to moderate inflammatory process. To evaluate the values we used the Student’s t-test, which found no statistically significant differences (P=0.064), however, there was a tendency to increased inflammation in the RS/PD group.

## Discussion

Results of this study have shown that RS was an effective method for causing chronic stress in rats, measured with plasma corticosterone levels (ng/ml). Animals treated with RS had higher levels of circulating corticosterone, with significant differences between groups receiving environmental enrichment vs. those that did not. This parameter is widely used as a marker of physiological changes associated with the presence and intensity of stress (14). Glucose showed only increased values in the RS/PD group, with no significant trend, which is in contrast to studies in which this parameter is increased in groups receiving chronic stress (2). This discrepancy may be due to some extent to the methodology used.

With regard to the various histopathological features analyzed, there was an increased presence of 100% vasodilation in rats in the RS/PD group (treated with stress and periodontal disease) vs. 20% of the PD group (treated only with periodontal disease). The increase of vasodilation observed in this investigation is associated with clinical features evidenced by Lindhe and Karring (27), who explained that early inflammatory changes associated with periodontal disease are likely to be expressed in the dentogingival plexus with increased blood supply to the affected area. If the inflammation it perpetuates, local factors, risk factors and host susceptibility may be considered in periodontal tissue destruction.

Significant differences expected in other characteristics of the analysis were not observed; it is likely that trends become apparent with monitoring and observation over an extended time period.

One of the parameters used to evaluate the role of RS in the severity of periodontal disease was the observation of the inflammatory infiltrate using the scale used by Liu *et al* (24), which according to the severity of inflammation, was: 0 for no inflammation; 1, mild; 2, moderate and 3, for severe. Rats in the RS/PD group had an average value of 2.8 inflammatory infiltrate corresponding to a moderate to severe inflammatory process, compared with the PD group where a value of 1.8 shows a mild to moderate inflammation. Therefore, RS modulates the inflammatory process in gingival tissue in rats treated with periodontal disease. These findings are consistent with those obtained by Takada *et al* (2), where rats subjected to restriction of movement with periodontal disease had a higher presence of inflammatory infiltrate, vasodilation and disorganization of connective tissue fibers than untreated subjects. Furthermore, Peruzzo *et al* (3) noted that restricting movement increased the expression of inflammatory factors and resorption in periodontal tissues in rats. Therefore, rats subjected to restriction of movement would have produced a greater inflammatory process vs. the untreated animals.

With regard to the degree of inflammation in the inter-radicular bone tissue, the rats in the RS/PD group had an average value of inflammatory infiltrate in the bone tissue of 5.5, corresponding to a moderate to severe inflammation vs. the PD group, which presented a value of 3.4, corresponding to a mild to moderate inflammation. Thus, RS also modulates the progress of the inflammatory process in bone tissue in rats treated with periodontal disease, although this difference was not statistically significant.

Consequently, RS would influence inflammatory processes in the gingival tissue and bone, but only in samples with periodontal disease (molars using a nylon ligature). However, RS by itself is unable to produce a more severe inflammatory process. This is consistent with Gaspersic *et al* (1), who suggested that stress by itself does not cause periodontal disease (no cause-effect). However, only when periodontal disease is present, stress may play a role, causing accelerated degradation of periodontal tissues, thus, creating a correlation between increased severity parameters and the presence of elevated levels of corticosterone.

The methodology used in this study shows that chronic RS increases the severity of inflammation, in the gingival tissue and bone. This finding is based on evidence obtained by conventional histopathological analysis only, which represents a limitation. For future studies, it would be necessary to evaluate the role of chronic stress on the severity of destructive processes in bone tissue, increasing the duration of the pilot phase and using other advanced histological techniques, since in this model it was not possible to measure bone destruction. Another alternative for these limitations is that repeated stimuli may generate a reduction in physiological responses elicited by exposure to a repeated homotypic (same) stressor, a phenomenon known as habituation (18). This possibility was considered in the study design, and to prevent it, we used a method of inducing high-intensity stress, accompanied by an interval between cycles and different environments. The influence of habituation cannot be ruled out, which may represent a limitation to our study. Nevertheless, the results of the present study showed that RS modulates periodontal inflammation and that the rat model described is suitable for investigating the association between stress and periodontal disease.

## Figures and Tables

**Table I:** Corticosterone and glucose plasma levels (mean ± SD) before and after restraint cycles.

	Corticosterone levels (ng/ml)		Glucose levels (mmol/l)	
Groups	Initial	Final	Initial	Final
RS/PD	195.1±71.5	348.2±135.4^a^	4.4±2.0	18.8±6.0
PD	186.2±55.4	232.0±69.2	2.8±2.3	13.5±7.3
RS	205.0±141.0	329.5±120.1^a^	4.1±2.2	13.5±2.4
Control	200.3±74.2	169.7±6.3	3.9±3,0	11.2±7.0

a Significant difference with PD, control. Mann-Whitney U test, statistical significance at P≤0.05. RS, restraint stress; PD, periodontal disease.
